# Effect of Cold Stabilization Duration on Organic Acids and Aroma Compounds during *Vitis vinifera* L. cv. Riesling Wine Bottle Storage

**DOI:** 10.3390/foods11091179

**Published:** 2022-04-19

**Authors:** Nongyu Xia, Haotian Cheng, Xuechen Yao, Qiuhong Pan, Nan Meng, Qingquan Yu

**Affiliations:** 1 Center for Viticulture & Enology, College of Food Science and Nutritional Engineering, China Agricultural University, Beijing 100083, China; xiany@cau.edu.cn (N.X.); chenght1995@163.com (H.C.); yaoxch@cau.edu.cn (X.Y.); 2 Department of Key Laboratory of Brewing Molecular Engineering of China Light Industry, Beijing Technology and Business University, Beijing 100048, China; 3 COFCO Chateau SunGod Greatwall, Zhangjiakou 075000, China; yuqq@cofco.com

**Keywords:** cold stabilization, aroma, organic acid, bottle storage, white wine

## Abstract

During the storage of wines in bottles, especially white wines, tartrate crystallization often occurs, which reduces the commercial value of the wines and therefore needs to be avoided by performing cold stabilization treatments before bottling. However, whether different cold treatment durations impact the quality of a wine’s aroma has not yet been of special concern. This research was conducted at an industrial scale to explore how cold treatments at −5.3 °C for 10 to 15 days impact the organic acids, aroma compounds, and sensory quality of Riesling dry white wines, and the variation was documented at the end of treatment, and at 6 and 12 months of bottle storage. The results showed that cold treatments significantly reduced tartaric acid concentrations and significantly affected the concentrations of most aroma components in the wines only after 12 months of bottle storage, including the main components of esters, norisoprenoids, terpenoids, and furfural. Moreover, the concentrations of some components showed an increasing trend with the bottle storage, especially 1,1,6-trimethyl-1,2-dihydronaphthalene (TDN), the characteristic volatile of Riesling wine, suggesting that an acidic condition resulting from cold treatment might facilitate the conversion of some aroma precursors into volatiles. In conclusion, cold stabilization treatments, within limits, can improve tartaric acid stability and could promote the conservation of aroma compounds during bottle storage without adversely affecting the aroma profile of the wines.

## 1. Introduction

Tartaric acid and malic acid are the primary organic acids in grape berries. The concentration of malic acid in wines is reduced during malolactic fermentation because of the ability of lactic acid bacteria to metabolize it into lactic acid. However, tartaric acid can maintain a relatively constant concentration during alcohol and malolactic fermentation because of its microbial stability [1]. The solubility of tartaric acid is usually reduced by low temperature, and then the tartaric acid precipitates as potassium bitartrate crystals [2]. This is a common phenomenon during the bottle storage of wines. Generally speaking, this precipitation does not affect the safety of wine. However, the broken-glass-like appearance leaves an unsatisfactory impression on consumers and reduces the commercial value of the wine. Therefore, some processes are often applied in the winery to avoid the formation of tartaric precipitation and improve tartaric stability [3].

The main components of tartrate precipitation are potassium hydrogen tartrate (KHT) and a small amount of calcium tartrate (CaT) [4,5]. The formation of tartaric precipitation includes three stages: the solution reaching supersaturation, crystal nucleation, and crystal growth [6,7]. During the bottle storage of wines, due to the presence of ethanol and the low temperature, the solubility of KHT decreases and tiny crystal embryos grow from the supersaturated solution [8]. After further nucleation to produce crystal nuclei, the ions in the solution continue to bind to the crystal nucleus, and the crystals continue to grow. Finally, the visible tartaric crystal is formed [7,9]. Besides ethanol and temperature, the wine compositions also affect the crystallization of the tartaric precipitation. Macromolecular compounds, such as the polyphenol rich in red wines and the mannoprotein rich in sparkling wines, can be adsorbed onto the growth site of the crystal surface through the ionic bond, hydrogen bond, or charge-transfer interaction to inhibit the crystallization formation. Macromolecular compounds are fewer in white wines, so white wines are more prone to tartaric precipitation [10]. This means the tartaric stabilization process is essential to produce white wines.

At present, wineries improve tartaric stability by reducing ion concentration and inhibiting crystal growth. The former methods include cold stabilization, ion exchange treatment, and electrodialysis. The latter involves adding polysaccharides (hydroxymethyl cellulose, gum arabic, mannoprotein, etc.), peptides (polyaspartic acid), or metatartaric acid (MTA) into wines to inhibit the formation of precipitation, or to modify their properties to be soluble at low temperature [2,11]. Correspondingly, many technologies have been developed [12,13]. The effects of ion-exchange treatment and electrodialysis on wine color, tannin content [4], anthocyanins, and other flavor compounds have been investigated in some research [9]. As for additives that have been studied more in recent years, the studies mainly focused on the effects of different additives [14,15], dosages and temperatures [8], structural characteristics [16], and states (solid or liquid) [14]. However, the additive dosage has been found to be a challenge to determine. Moreover, the legality of applying additives in wine production varies from country to country [9]. Although the International Organisation of Vine and Wine (OIV) has approved the additives mentioned above [17], they are not yet allowed in China [18]. Therefore, the application of additives is not the first choice in production in China. In contrast, although the most traditional cold stabilization treatment takes a long time, it was found to be the most economical approach and wildly used because of its simple operation, the versatility of its equipment, the recyclability of energy during cooling, and the convenience for the batch processing of wine [12].

Cold stabilization treatment is performed by storing wines for a period at a temperature close to the freezing point, where the tartrate solution is more likely to reach supersaturation and promote the formation of KHT precipitation [2]. The freezing point is obtained by the calculation: alcohol content minus one and then divided by two [2]. The duration of the cold treatment is based on the experience of the wine producers. Unlike phenol compounds [16,19], the changes of aroma compounds in wines with cold treatment are little noticed. However, aroma quality is an important indicator for evaluating the quality of wines. The aroma compounds in wines can mainly provide fruity, floral, honey, herbaceous, toasty, and kerosene aromas, and the uniqueness of the aroma profile of wines depends on the composition and concentrations of the aroma compounds [20,21,22].

This study investigated the effects of cold stabilization duration and bottle-storage duration on the concentrations of organic acids and aroma compounds in the *Vitis vinifera* L. cv. Riesling dry white wines produced in the COFCO Chateau SunGod GreatWall in China. This winery performs traditional cold stabilization treatment for 15 days on white wines. Based on this, this present experiment applied 10 to 15 days of cold stabilization. The objective was to explore whether and how different treatment durations affect wine qualities involving the organic acid and aroma compounds during bottle storage. The results of this research would help wineries know more about the quality change caused by cold stabilization and bottle storage.

## 2. Materials and Methods

### 2.1. Description of Winemaking

**Figure 1:**
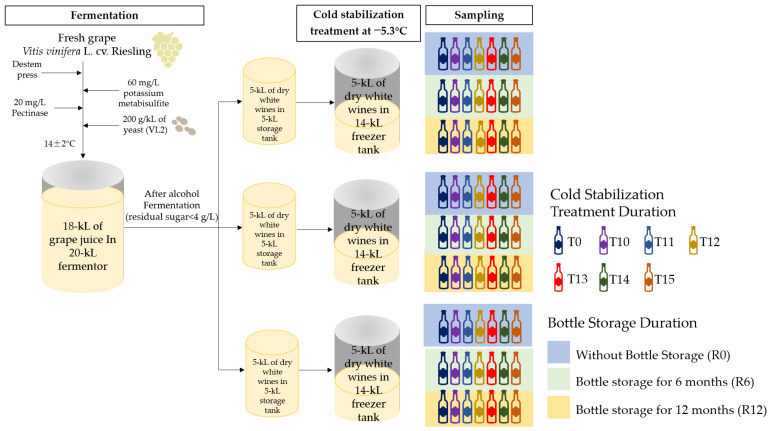
Schematic diagram of winemaking and experiments.

The dry white wines used in this experiment were produced in October 2017 in the COFCO Chateau SunGod GreatWall (40°20′56″ N, 115°33′15″ E), Huailai county, Hebei province. The grape variety used for winemaking was *Vitis vinifera* L. cv. Riesling grafted on a 5BB rootstock and cultivated in 1979. The process complied with the standard procedures of the winery. Grape clusters at commercial maturity were harvested by hand. The clusters were transferred quickly to the workshop and destemmed. Then, the destemmed grapes were pressed by a pneumatic press. About 18 kiloliters (kL) of grape juice (201.1 g/L total sugar, titratable acidity of 8.8, and pH 3.27) was obtained and 60 mg/L potassium metabisulfite was added. After clarification by adding 20 mg/L pectinase and ranking, the obtained clean juice was used for fermentation in a 20 kL fermenter. A total of 200 g/kL of VL2 yeast was inoculated in the grape juice. The fermentation temperature was controlled at 14 ± 2 °C. After the alcohol fermentation ended, potassium metabisulfite was added to adjust the free SO_2_ concentration, and malolactic fermentation was not undertaken. The general oenological parameters of the wines were measured using the methods according to the “Analytical methods of wine and fruit wine”, which is the national standard of China (GB/T 15038-2006) [23]. The free and total SO_2_ were measured by an aeration/oxidation method. The residual sugar was detected by titration with Fehling’s reagent. The alcohol content was determined by the distillation/densimetry method. The parameters in the fresh wine were as follows: alcohol content of 11.6% (*v*/*v*), residual sugar content of 2.5 g/L, and free and total concentrations of SO_2_ of 29 and 101 mg/L, respectively. Subsequently, the wine was transferred into three 5 kL stainless steel tanks and stored on lees. The headspaces of the tanks were filled with nitrogen to keep the wines from oxidation. These tanks were placed in a constant 17 ± 2 °C temperature workshop. Free SO_2_ was measured once a month and it was ensured that free SO_2_ was maintained at 25–30 mg/L by timely adding potassium metabisulfite to protect the wines from microbial deterioration (Figure 1).

### 2.2. Tartaric Stabilization Treatment

Cold stabilization treatment was carried out before the bottling process of the winery in May of 2019. First, the wines in the different 5 kL stainless steel tanks were transferred to three 14 kL freezer tanks (Shijiazhuang Heng Chang Food Packaging Machinery Co., Ltd., Shijiazhuang, China) and stirred, which were also taken as three replicates (Figure 1). The refrigerant of the freezer tanks was 33–35% (*v*/*v*) industrial alcohol. The basic physico-chemical indicators of the wines were as follows: a pH value of 3.12 ± 0.21, alcohol content of 11.6 ± 0.13% (*v*/*v*), residual sugar content of 2.5 ± 0.85 g/L, total acidity of 6.7 ± 0.23 g/L as tartaric acid, and free and total sulfur of 27 ± 3.75 mg/L and 112 ± 2.42 mg/L, respectively. The freezing temperature was set at about −5.3 °C according to the calculation of the freezing point [2].

### 2.3. Rapid Discrimination of Tartaric Stability

A total of 200 mL of the uniformly stirred wine per tank was taken out and immediately filtered with a 0.45 μm filter membrane (Tianjin jinteng Experimental Equipment Co., Ltd.; Tianjin, China) by a diaphragm vacuum pump (GM-0.33A; Tianjin jinteng Experimental Equipment Co., Ltd.; Tianjin, China). The filtrate was loaded into an Erlenmeyer flask, then capped with a rubber stopper and placed in a −18 °C freezer for 24 h. After that, the wine filtrate was thawed and observed with the naked eye to judge whether there was precipitation in the wine. The wine without any sediment was considered to have reached tartaric stability.

### 2.4. Experiment Design and Sampling

According to the standard procedures of the winery, the cold stabilization treatment of dry white wine should last for 15 days. In this study, the sampling was conducted before cold stabilization and at 10, 11, 12, 13, 14, and 15 days of cold treatment (respectively marked as T10, T11, T12 T13, T14, and T15). To avoid the redissolution of the tartaric precipitation, the wine sample was collected in a one-liter thermos flask and was quickly filtered with the diaphragm vacuum pump and a 0.45 μm filter membrane. Then, we sampled 750 mL of wines by a one-liter cylinder and immediately poured them into the standard green glass bottles. After being manually sealed with natural corks, the bottle mouths were wrapped with shrinkable polyvinyl chloride (PVC) capsules. Then, the samples were all laid down to minimize the infiltration of external oxygen and stored in the wine cellar. The cellar temperature was kept at 14–16 °C all year round, and the relative air humidity was about 70%. A total of 63 bottles of wines were used for this study and three bottles were taken out per treatment group after cold treatment, and at 6 months and 12 months of bottle storage, respectively (Figure 1). Every bottle of the sample was applied in the determination of organic acid, aroma compounds, and sensory evaluation.

### 2.5. Determination of Organic Acids and pH

In the wines, the pH was measured by PB-10 m (Sartorius, Germany). The detection of organic acids, including tartaric acid, citric acid, lactic acid, malic acid, acetic acid, and succinic acid, was slightly modified according to the previous method described by Liu et al. [24]. The wine samples were filtered through a 0.22 μm pore polyethersulfone filter membrane (Tianjin Jinteng Experimental Equipment Co., Ltd.; Tianjin, China) and determined by Agilent 1200 high-performance liquid chromatography with an ultraviolet detector using an HPX-87H Aminex ion-exchange column (300 mm × 7.8 mm, Bio-Rad Laboratories, Hercules, CA, USA). The detection parameters are as follows: the injection volume of samples 20 µL, column temperature of 60 °C, a mobile phase flow rate (5 mmol/L H_2_SO_4_ in water) of 0.6 mL/min, and a detection wavelength of 280 nm. Organic acids were identified according to the retention time of the organic acid standard and quantified based on the standard curves.

### 2.6. Qualification of Aroma Compounds

The aroma compounds in wines were detected according to the previously published method by using a headspace solid-phase microextraction (HS-SPME) equipped with 2 cm DVB/CAR/PDMS 50/30 µm SPME fiber (Supelco, Bellefonte, PA, USA), and an Agilent 7890 gas chromatography–5975 mass spectrometer (Agilent Technologies, Santa Clara, CA, USA) [25,26]. The wine sample of 5 mL was mixed with 1.00 ± 0.01 g NaCl (to promote the volatilization of the compound) and 10 μL 4-methyl-2-pentanol (internal standard, 1.0006 g/L) in a 20 mL autosampler vial. Then the vial was immediately capped tightly with a PTFE–silicon septum and put in the CTC CombiPAL autosampler (CTC Analytics, Zwingen, Switzerland). After the sample in the vial was heated at 40 °C for 30 min, the activated SPME fiber was automatically inserted into the headspace of the vial, to be continually slightly shaken for 30 min at 40 °C to assure the distribution of aroma compounds in the three phases (wine sample, headspace, and SPME fiber) to reach equilibrium. After that, the fiber was desorbed by being inserted into the GC injector under splitless mode at 250 °C for 8 min. The aroma compounds were separated on an HP-INNOWAX capillary column (60 m × 0.25 mm × 0.25 µm, J&W Scientific, Folsom, CA, USA) with carrier gas of high-purity helium (He > 99.999%) with a 1 mL/min flow rate. The column oven first held an initial temperature of 50 °C for 1 min, then heated up at the rate of 3 °C/min to 220 °C and held for 5 min. The mass spectrum applied electron ionization (EI) at 70 eV energy to ionize the molecules of aroma compounds, the ion source and quadrupole temperatures were 250 °C and 150 °C, respectively. The mode of the mass detector was a full scan (*m*/*z* 30–350).

The aroma compound identification was completed using the automatic mass spectral deconvolution and identification system (AMDIS) and NIST 11 database based on the retention indices calculated by C7–C24 n-alkane series (Supelco), retention time, and mass spectrum of volatile compounds. The peak areas were calculated using Chemstation software (Agilent Technologies, Santa Clara, CA, USA). Then, the peak area ratio for each volatile compound to the internal standard was brought into the corresponding standard curve to calculate the concentration. For compounds with and without available standards, the calibration curve of standards and the standards with similar chemical structure, functional group or similar carbon number for quantification, respectively, were used. A synthetic matrix containing 2 g/L glucose, 7 g/L tartaric acid, and 13% alcohol (*v*/*v*) with a pH of 3.5 was prepared, then standards were mixed into it and it was diluted into 10 successive levels.

### 2.7. Calculation of Odor Activity Value (OAV)

The OAV of the aroma compounds was calculated by its concentration divided by its perception threshold [25]. The OAVs of the compounds that contribute to the same aroma type were added up to illustrate the potential change of the four aroma types in the present wine (fruity, floral, kerosene, and honey odor). Since the matrix of samples could interfere with the detection thresholds of the aroma compounds, the OAVs here were only used to compare the aroma profile differences in different treatment wines at different bottle storage durations within this trial.

### 2.8. Aroma Sensory Evaluation

#### 2.8.1. General Conditions

The sensory evaluations were conducted in a professional sensory laboratory equipped with six individual booths under a controlled room temperature (20 °C), white lighting, and airflow conditions at 6 and 12 months of bottle storage, respectively. The wine-tasting glasses of the International Standards Organization (ISO 3591:1977) with caps were used in the process of tasting.

#### 2.8.2. Panel Training and Sensory Evaluation

The sensory evaluation panel was composed of five professional winetasters who have trained regularly and obtained the highest-class National Winetaster Certificates, two males and three females aged 25 to 40 years old. All panelists attended two 1 h training sessions. In the first session, sample wines of each treatment were evaluated and the attributes (fruity, floral, honey, and kerosene) of the samples were obtained. In the second session, five different Riesling dry white wines from the Chateau Sungod winery were used to conduct the intensity training. In the sensory evaluation, a Riesling dry wine from the winery was randomly selected and scored as a reference wine to unify the scoring standard before the sensory evaluation. Each tasting glass was coded with random three-digit numbers and served with approximately 30 mL of wine at 12 °C. Three bottles of each treatment wine were used in evaluations in three tasting sessions to ensure each treatment sample could be tasted three times in duplicate. A two-minute break was required between samples. Quantitative descriptive analysis (QDA) was performed to evaluate the fruity, floral, honey, kerosene, and overall aroma intensity of the experimental wine samples (out of 10 points); 0 points express that the aroma was not perceptible at all, and 10 points indicate that the aroma was strongly perceptible. PanelCheck v1.4.2 software (www.panelcheck.com accessed on 16 March 2022) was used to assess the discrimination of the sensory evaluation by *F* plot (*p* < 0.05) and estimate the repeatability for each assessor by *MSE* plot based on one-way ANOVA [27,28]. The sensory data were statistically analyzed by one-way ANOVA (Duncan, *p* < 0.05).

### 2.9. Statistical Analyses

One-way ANOVA (Duncan, *p* < 0.05) was performed by using the ‘agricolae’ package in R statistical environment (3.6.2). Two-way ANOVA was conducted by IBM SPSS Statistics (version 25). *K*-means algorithm analysis was applied by using the ‘fviz_nbclust’ function in the ‘factoextra’ package in R statistical environment (3.6.2). Correlation analyses were conducted using the ‘Corrplot’ package in R statistical environment (3.6.2). All graphs were finished by using the ‘ggplot2’ package in R statistical environment (3.6.2).

## 3. Results

### 3.1. Tartaric Stability and Tartaric Acid Reduction

As described in the Materials and Methods section, tartaric stability was tested by a rapid discrimination method. Tartaric precipitation was not observed in the treated wines starting from the 13-day cold treatment, suggesting that the wines had gained tartaric stability. Compared with the traditional 15-day cold stabilization treatment in the wineries, this can save two days. The pH value of wines did not change significantly at the end of cold treatment, but compared with the control group, the pH values presented a decline in the cold treatment groups during subsequent bottle storage durations (Table 1), and the longer the bottle storage duration, the lower the pH values.

One-way and two-way ANOVA were carried out, and the results showed that compared with the control group without cold treatment, cold stabilization treatments for 10–15 days (expressed as T10–T15) reduced tartaric acid and succinic acid concentrations significantly by approximately 10.85–15.97% and 11.32–24.12%, respectively. As expected, there was no significant variation between different treatment groups on citric acid and malic acid concentrations during the whole bottle-storage duration. Concerning acetic acid and lactic acid, the effect of treatments appeared to be temporary and inconsistent before and after bottle storage (Table 2).

During the entire bottle storage, the concentrations of tartaric acid, citric acid, and lactic acid remained stable. The concentration of malic acid in most treatment groups, except for T12 and T15, and the concentration of succinic acid in all groups decreased significantly after bottle storage. In contrast, the concentration of acetic acid in all treatment groups significantly increased after bottle storage.

The results of the two-way ANOVA analyses showed that only the concentrations of acetic acid and succinic acid were significantly affected by the interaction of cold stabilization duration and bottle-storage duration, whose concentrations were relatively lower than those of other organic acids in the wines.

### 3.2. Changes of Aroma Compounds

**Figure 2:**
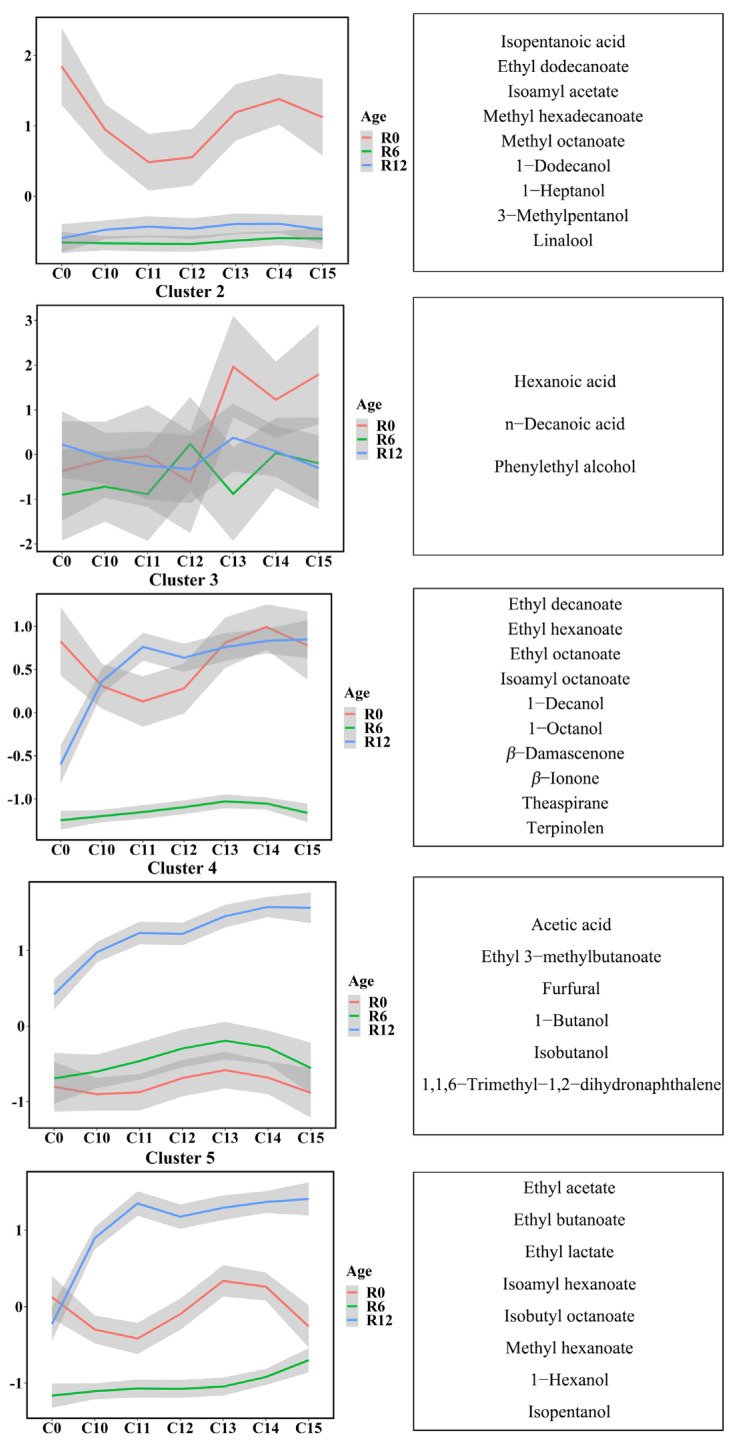
*K*-means clustering analysis based on the normalized concentrations (left) and the corresponding aroma compounds in each cluster (right) in Riesling wines. “R0” represents before bottling, and “R6” and “R12” individually represent bottle storage for 6 months and 12 months; “T0” represents the control without any cold treatment, and “T10 to T15” individually represent cold treatment for 10 to 15 days. The vertical coordinate was the best value for each center point of the cluster calculated by the *k*-means algorithm.

To understand an overall variation of the aroma compounds in the Riesling wines with and without cold stabilization treatment during bottle storage, we performed a k-means clustering analysis based on the normalized concentration (Figure 2). We detected 36 aroma compounds clustered into five clusters according to their variation. The components corresponding to each cluster and their concentrations were listed in Table S1. The one-way and two-way ANOVA involving bottle storage duration and cold treatment as factors were conducted to further explore the significant differences in aroma compounds among samples (Table S1).

Overall, the aroma components in cluster 2 had a higher concentration in the wines with cold treatment for over 13 days than in other treatments and control wines at the end of treatment (marked as R0). This effect disappeared during subsequent bottle storage (R6 and R12). The compounds were phenylethyl alcohol, hexanoic acid, and *n*-decanoic acid. Except that, the compounds in cluster 1, cluster 3, and cluster 5 were slightly reduced only in the wines (R0) with 11-day (and 12-day) cold treatment, and the concentrations of compounds in cluster 4 were not changed by cold treatment (Figure 2).

As shown in Table S1, during bottle storage, the concentrations of the compounds in cluster 1 decreased significantly in all wines after 6 months of bottle storage, but no significant difference was found between 6-month and 12-month bottle-storage wines. Among them, linalool and isoamyl acetate were the main terpenoid and acetate ester in the Riesling white wines, respectively. It was also noticed that the concentrations of the compounds in cluster 4 (such as 1,1,6-Trimethyl-1,2-dihydronaphthalene (TDN), furfural, etc.) significantly increased after 12 months of bottling storage. As for the concentrations of the compounds in cluster 3 and cluster 5, the change with the bottle-storage duration differed between the control group and cold treatment groups. The concentrations of cluster 3 compounds in the T0 group decreased significantly after bottle storage, while almost no significant difference was observed in the T10–T15 groups. Cluster 5 included the main component of acetates (ethyl acetate), fatty acid ethyl esters (ethyl lactate), and higher alcohols (isopentanol). Compared with the wine before storage, the concentrations of aroma compounds in the T0 groups did not change significantly during the subsequent bottle storage, while those of the T10–T15 groups increased after 12-month bottle storage.

Interestingly, compared with the control group, we observed that most compounds did not change significantly because of the cold treatments at 0 months and 6 months after bottle storage, while some compounds in cluster 1 (1-heptanol, isoamyl acetate, linalool, and methyl octanoate), some of cluster 4 (1-butanol and acetic acid), most of the cluster 3 compounds (such as *β*-damascenone, *β*-ionone, ethyl hexanoate, ethyl octanoate, etc.), all the cluster 5 compounds (such as isopentanol, ethyl lactate, ethyl acetate, ethyl butanoate, etc.) showed significantly higher concentrations in the T10–T15 groups after 12 months of bottle storage. These compounds mainly belonged to ethyl esters, higher alcohols, norisoprenoids, and terpenoids. As for cluster 4, only some treatment groups significantly changed the concentrations of some compounds during the bottle storage duration; for example, the concentrations of TDN in the T14 and T15 groups increased at the end of treatment (R0), and increased in the T12–T15 groups at 6 months of bottle storage (R6), but these differences disappeared at 12 months of bottle storage.

The interaction of cold stabilization duration and bottle-storage duration significantly influenced the concentrations of most compounds in the wines, except two compounds in cluster 1 (3-methylpentanol and isopentanoic acid), *β*-ionone in cluster 3, and three compounds (ethyl 3-methylbutanoate, TDN, and furfural) in cluster 4.

### 3.3. Effect of Wine pH Reduction on Aroma Compounds and the Correlation between Aroma Compounds

The wine pH value gradually reduced during bottle storage (Table 1). To understand whether the variation of the aroma compounds with the cold stabilization treatment is related to the decline of the wine pH value, the correlation analyses were conducted. In addition, the relationships among the changes of the concentrations of various compounds were explored. The correlation coefficient *r*-values and *p*-values of the correlation analyses were all further shown in Table S2.

**Figure 3:**
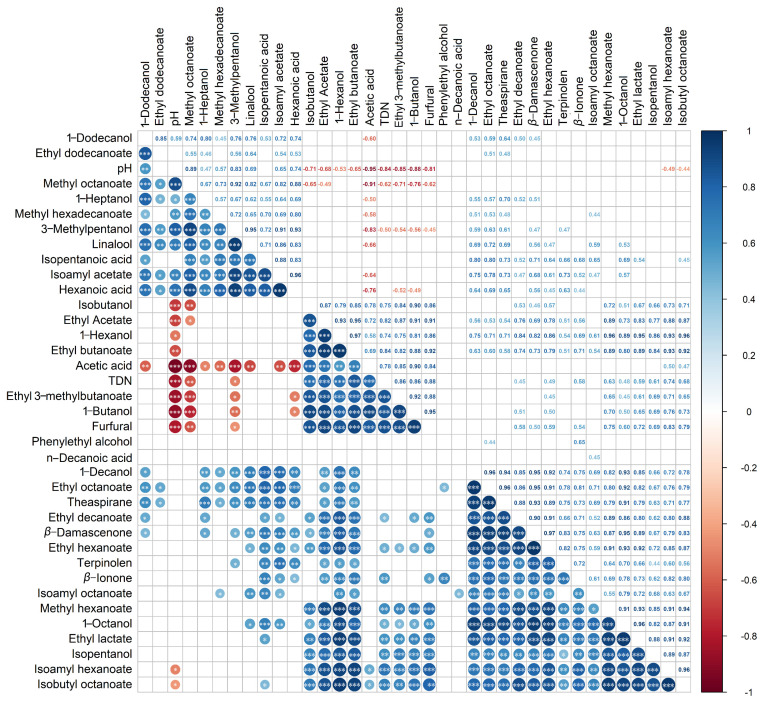
Correlation analyses between the pH value and the concentration of individual aroma compounds and between different aroma compounds. The “*”, “**”, and “***” represented that the correlations were statistically significant, which means *p* < 0.05, *p* < 0.01, *p* < 0.001, respectively.

From Figure 3, it was observed that some compounds had a significantly negative correlation with the pH value, such as some fatty acid esters (ethyl butanoate, ethyl 3-methylbutanoate), the main component of acetate esters (ethyl acetate), higher alcohols (1-hexanol and isobutanol,1-butanol), volatile acids (acetic acid), norisoprenoids (TDN), and furfural, indicated that the lower pH resulting from the cold treatments could promote the formation of them. In contrast, linalool, three esters less than 10 µg/L (methyl octanoate, isoamyl acetate, methyl hexadecanoate), 1-heptanol, 3-methylpentanol, and hexanoic acid had significantly positive correlations with the pH value, which was verified by the lower concentrations of these components after bottle storage and cold stabilization treatments. Besides those, isoamyl hexanoate and isoamyl octanoate were significantly positively correlated with their substrates, isopentanol, for esterification reaction. The limited increase of these two esters could result from the low concentrations or absence of corresponding acids (hexanoic acid and octanoic acid). Isoamyl acetate, which decreased significantly after bottle storage, showed a significant negative correlation with acetic acid, which increased steadily, suggesting that the hydrolysis of isoamyl acetate at low pH conditions might release the acetic acid.

### 3.4. Aroma Description Based on Odor Activity Values (OAV) and Sensory Evaluation

**Figure 4:**
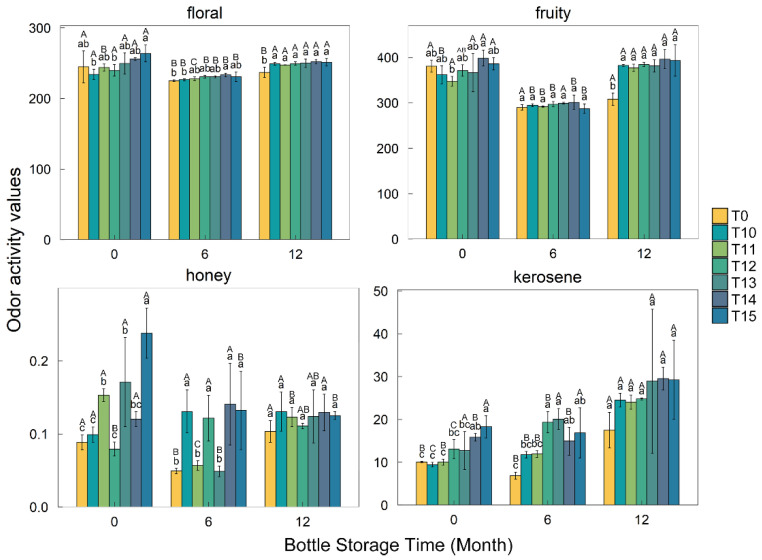
Effect of cold treatment duration on the OAVs of four aroma types (floral, fruity, honey, and kerosene). Samples (*n* = 3). Different lowercase letters represent significant differences in the OAV between different groups at the same bottle-storage period. Different capital letters represent significant differences in the OAV between different bottle-storage durations for the same treatment group (Duncan, *p* < 0.05).

The types of aromas and the OAVs contributed by each compound were listed in Table S3. The OAVs of the different compounds that contribute the same odor description were summed up. Herein, the aroma variation of the wines was mainly concerned with fruity, floral, kerosene, and honey smells (Figure 4).

**Figure 5:**
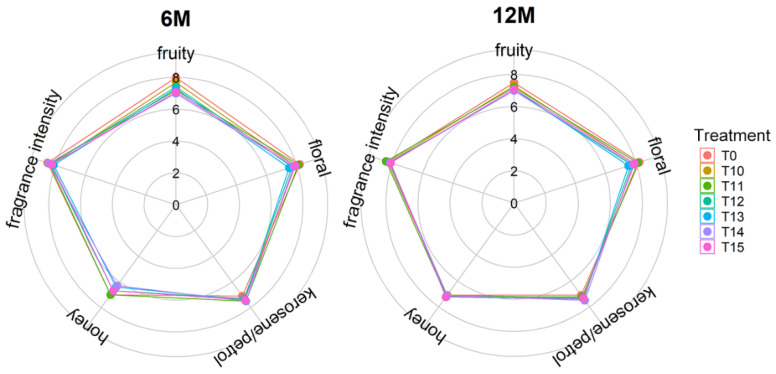
Aroma sensory evaluation scores of the 11-day to 15-day cold treatment wines (T11–T15) and the control wine (T0) at 6 months and 12 months of bottle storage.

At 0 and 6 months of bottle storage, the OAVs of both the fruity and floral smells had no significant difference between the cold treatment wines and the control group except for the floral OAV at 6 months of bottle storage. After bottle storage for 12 months, all cold treatments significantly increased the fruity and floral OAVs, which was related to the increase in the concentrations of ethyl esters, *β*-damascenone, *β*-ionone, and others (Table S1). These compounds provide the fruity and floral aroma [20]. Compared with the control group without cold treatment, the OAV corresponding to the kerosene smell was significantly increased in the T14 and T15 wines at the end of the cold treatment (R0), and in the wines with cold treatments over 12 days (T12–T15) after bottle storage for 6 months, respectively. Moreover, the OAV of kerosene steadily increased during bottle storage and no statistical difference existed among the treated wines at the 12-month point of bottle storage, which was closely related to the increase of TDN concentration (Table S1). The kerosene was provided by TDN in Riesling wines [22]. The honey smell is characterized by phenylethyl alcohol in this study [20]. The OAV corresponding to the honey smell increased in the T11, T13, and T15 wines at the end of cold treatment (R0), and in the cold treatment wines, except for T11 and T13, 6 months after bottle storage. But at 12 months of bottle storage, these differences all disappeared, even though the OAV corresponding to the honey aroma was too low, so the variation did not influence the wine aroma sensory characteristics. Regarding aroma sensory evaluation, various aroma characteristics did not show statistical differences between the different groups at the 6-month and 12-month points of bottle storage (Figure 5).

## 4. Discussion

In this study, three main results were received. First, the concentration of tartaric acid in the wines declined with cold stabilization treatment from 11 days to 15 days, as expected. Second, the wine pH value declined significantly during bottle storage, and cold treatments significantly reduced the pH value of wines compared with the control group after 6 and 12 months of bottle storage. Finally, some main components of ethyl esters and norisoprenoids tend to increase during bottle storage, which is highly correlated to the pH declining.

The cold treatment duration for the wines to obtain tartaric stability varies with wines [2]. In 1999, Vernhet et al. studied the composition of the tartrate precipitates deposited on stainless steel tanks during the cold stabilization of white wines. They found that a complete stabilization of Grenache white wine was achieved within eight days, whereas the Maccabeu white wine had not yet achieved stabilization after six weeks [10]. Researchers compared the effects of cold treatment, ion exchange resins, electrodialysis, potassium polyaspartate, and carboxymethyl cellulose on the tartrate stabilization of Monastrell red wines. They concluded that cold treatment for 15 days could effectively keep the tartrate stability of wines even after one year of bottle storage [13]. Benítez et al. observed that cold treatment for seven days could impart tartaric stabilization to fino, medium, and cream wines [15]. In our study, no new crystal precipitation in the Riesling dry wines appeared in the cold treatment at −5.3 °C for over 13 days.

Potassium ion (K^+^) is at higher concentrations in grape juice and the bitartrate (HT^−^) species is usually at higher concentrations than tartrate (T^2−^) at the normal wine pH range (pH 3.0–4.0) [5]. As a result, wine cold stabilization treatment primarily generates potassium bitartrate (KHT) crystals [5]. KHT precipitation is always accompanied by an increase or decrease in wine pH, which highly depends on the pH value prior to precipitation [2], and pH 3. 65 is a key dividing point [5]. That is, tartrate stabilization treatment on wines below pH 3.65 may result in a pH decrease because the wines have a much higher concentration of free tartaric acid (H_2_T) in comparison to its tartrate (T^2−^), and H_2_T/HT^−^ equilibrium shifts to increase HT^−^ and liberate H^+^, which eventually leads to a pH decrease [2]. In contrast, in the wines above pH 3.65, KHT precipitation will result in a pH increase as a higher concentration of T^2−^ makes the HT^−^/T^2−^ equilibrium shift to increase HT^−^ through bounding H^+^ [5]. The Riesling wine pH was about 3.16 before the cold treatments in this study. The above tartaric acid chemistry explains why the pH declined with the cold treatment duration and bottle storage.

Much research so far has focused on the effect of the cold stabilization treatments such as ion exchange resins, carboxymethylcellulose, potassium polyaspartate, and electrodialysis on the phenolic profile and chromatic characteristics [13,16,29]. Mislata et al. observed that cation-exchange resin-treated wines had higher concentrations of aromatic compounds such as hexyl acetate, isobutanol, 2-phenylethyl alcohol, ethyl isovalerate, and diethyl succinate after 6 months of bottle storage [4].

Esters are important contributors to wine aroma [30]. During bottle storage, esterification and ester hydrolysis strongly depend on wine pH [31]. Lower pH will accelerate the acid-catalyzed esterification or hydrolysis [31,32,33]. Our result also demonstrated this point to some extent. In this study, apart from methyl hexadecanoate, methyl octanoate, and ethyl dodecanoate, other esters detected in the Riesling wines all showed increases during 6 to 12 months of bottle storage. However, only the increase in the concentrations of ethyl butanoate, ethyl 3-methylbutanoate, and ethyl acetate was closely correlated to the decrease in pH, whose OAV and concentrations were small in wines. The changes of other esters that had significantly higher concentrations, such as ethyl lactate and ethyl hexanoate, were not significantly correlated with the change in pH values. Therefore, the change of ethyl butanoate, ethyl 3-methylbutanoate, and ethyl acetate exerted less influence on the flavor quality of the wine, which may explain why there was no statistically significant difference in aroma sensory characteristics between different treatment groups and different bottle-storage durations.

It has been widely known that many wine odorants exist as glycosidically bound precursors that will convert into free, volatile forms via acid catalysis during storage, and lower pH could promote the hydrolysis of glycoside, as well as the isoprenoid rearrangements observed in the resulting aglycone intermediates [34]. TDN is considered a characteristic aroma component of Riesling wines, particularly aged Riesling [35]. This compound has an aroma reminiscent of “kerosene” and a sensory threshold of 2 μg/L [36]. In this study, TDN was present at a concentration over 100 μg/L and dominated the aroma of the wines. Previous studies have demonstrated that TDN can be generated by acid-catalyzed hydrolysis of Riesling acetal (an intermediate of C13-norisoprenoid glycoconjugates in grapes) during wine storage, and wines with lower pH usually have higher concentrations of TDN [35,36]. This phenomenon was also observed in our study, where the concentration of TDN was significantly negatively correlated with the pH value, and the concentration was increased with the cold stabilization treatment duration and storage duration.

In our study, furfural presented a similar variation trend with TDN, which is closely associated with the pH decline. Through acid-catalyzed degradation, furfural can be converted from pentoses in wine [37]. This result may indicate the existence of pentoses.

For linalool, same as the results of other research, linalool decreased after bottle storage for 12 months as the pH decreased, which might be caused by the acid-catalyzed transformations into furan linalool oxides [38,39].

## 5. Conclusions

In summary, cold treatment significantly reduced the concentration of tartaric acid and also lowered the pH value in the dry Riesling wines. The pH values also decreased along with the bottle-storage duration, proving that cold stabilization treatment could lead to lower pH in wine with an original pH of less than 3.65. Perhaps that was why the concentrations of most aroma compounds were varied by the cold treatment duration and bottle-storage duration, and by the interaction between them. Compared with the no cold stabilization treatment group (T0), most aroma compounds in the cold treatment groups showed higher concentrations at 12 months of bottle storage; in particular, the main components of esters, norisoprenoids, terpenoids, and furfural increased significantly. In addition, some components increased along with bottle storage, especially 1,1,6-trimethyl-1,2-dihydronaphthalene (TDN), the characteristic volatile of Riesling wine. It was suggested that the acid-dependent reactions during wine bottle-storage after cold stabilization could promote the change of these aroma compounds. However, from the perspective of sensory evaluation, the improvement was still limited.

## Figures and Tables

**Table 1:** Effects of cold treatment duration and bottle storage duration on wine pH value.

Cold Treatment Duration (Days)	Without Storage in Bottles	Bottle Storage for 6 Months	Bottle Storage for 12 Months
0	3.16 ± 0.01 aA	3.13 ± 0.02 aA	3.03 ± 0.02 aB
10	3.16 ± 0.01 aA	3.09 ± 0.01 bB	2.98 ± 0.01 bC
11	3.15 ± 0.01 aA	3.08 ± 0.02 bcB	2.99 ± 0.01 bC
12	3.17 ± 0.02 aA	3.09 ± 0.01 bB	3.01 ± 0.04 abC
13	3.17 ± 0.01 aA	3.04 ± 0.01 dB	2.99 ± 0.01 bC
14	3.15 ± 0.01 aA	3.05 ± 0.01 dB	2.99 ± 0.01 bC
15	3.15 ± 0.01 aA	3.06 ± 0.01 cdB	2.98 ± 0.02 bC

“Mean ± sd” (*n* = 3) were shown in the table. Different lowercase letters represent significant differences in the pH values between different groups at the same bottle-storage period. Different capital letters represent significant differences in the pH values among bottle-storage durations for the same treatment group (Duncan, *p* < 0.05).

**Table 2:** Effects of cold treatment duration and bottle-storage duration on concentrations of organic acids (unit: mg/L).

Bottle-Storage Duration (Months)	Cold Stabilization Duration (Days)	Tartaric Acid	Citric Acid	Lactic Acid	MALIC ACID	Acetic Acid	Succinic Acid
0	0	2149.93 ± 60.25 aA	140.17 ± 0.64 abA	845.20 ± 49.15 aA	1759.27 ± 9.87 aA	156.77 ± 4.45 aC	114.53 ± 2.65 aA
	10	1913.67 ± 74.63 bA	137.80 ± 1.80 bA	818.20 ± 28.69 abA	1769.77 ± 13.42 aA	141.37 ± 1.68 bC	97.20 ± 2.84 bA
	11	1858.20 ± 83.06 bA	139.13 ± 1.04 abA	792.20 ± 2.65 bcA	1778.43 ± 30.31 aA	141.00 ± 3.97 bC	99.86 ± 1.75 bA
	12	1838.87 ± 78.50 bA	139.13 ± 1.26 abA	788.53 ± 6.51 bcA	1779.10 ± 41.46 aA	142.10 ± 4.79 bC	99.65 ± 3.49 bA
	13	1833.93 ± 77.72 bA	138.63 ± 1.26 abA	756.87 ± 5.03 cB	1759.60 ± 16.46 aA	141.73 ± 2.29 bC	101.47 ± 1.74 bA
	14	1817.13 ± 72.51 bA	140.63 ± 1.15 aA	784.20 ± 1.00 bcB	1757.27 ± 15.14 aA	141.00 ± 4.79 bC	101.57 ± 0.81 bA
	15	1809.40 ± 72.72 bA	138.47 ± 1.53 bA	790.20 ± 40.04 bcA	1749.93 ± 21.05 aA	141.73 ± 7.32 bB	99.87 ± 3.25 bA
6	0	2131.93 ± 60.25 aA	139.97 ± 0.29 aA	806.20 ± 12.12 aA	1730.93 ± 7.89 aB	265.67 ± 24.60 dB	111.85 ± 1.32 aAB
	10	1895.67 ± 74.63 bA	139.13 ± 0.76 aA	789.53 ± 11.59 abA	1739.33 ± 10.74 aB	286.57 ± 19.81 cdB	84.87 ± 2.81 bB
	11	1840.20 ± 83.06 bA	139.63 ± 0.76 aA	791.87 ± 4.93 abA	1746.27 ± 24.25 aAB	311.50 ± 6.12 bcB	90.52 ± 4.57 bB
	12	1820.87 ± 78.50 bA	139.80 ± 1.80 aA	795.53 ± 9.50 abA	1746.80 ± 33.17 aA	315.90 ± 14.55 abB	89.10 ± 1.82 bB
	13	1815.93 ± 77.72 bA	138.97 ± 1.44 aA	797.53 ± 8.62 abA	1731.20 ± 13.17 aAB	324.70 ± 3.30 abB	88.00 ± 1.93 bB
	14	1799.13 ± 72.51 bA	140.13 ± 2.02 aA	783.87 ± 3.21 bB	1729.33 ± 12.12 aB	322.50 ± 6.12 abB	87.27 ± 5.30 bB
	15	1791.40 ± 72.72 bA	140.30 ± 2.60 aA	799.53 ± 9.81 abA	1723.47 ± 16.84 aA	342.30 ± 15.12 aA	89.53 ± 0.50 bB
12	0	2119.27 ± 63.22 aA	139.47 ± 1.53 aA	844.87 ± 34.65 aA	1733.47 ± 8.49 abB	326.17 ± 16.48 bA	109.64 ± 2.04 aB
	10	1889.27 ± 68.17 bA	138.30 ± 1.80 aA	801.87 ± 38.50 bA	1708.27 ± 5.06 cC	339.63 ± 5.44 abA	90.27 ± 4.54 bAB
	11	1849.13 ± 65.63 bA	139.13 ± 1.04 aA	796.87 ± 8.02 bA	1720.80 ± 9.83 bcB	347.10 ± 12.44 abA	84.15 ± 2.82 cB
	12	1819.67 ± 73.12 bA	139.63 ± 2.02 aA	799.87 ± 8.39 bA	1716.80 ± 10.79 bcA	359.83 ± 12.07 aA	83.57 ± 2.22 cC
	13	1798.47 ± 64.50 bA	139.13 ± 1.53 aA	791.20 ± 8.66 bA	1725.20 ± 18.76 abcB	357.73 ± 9.86 aA	87.10 ± 1.05 bcB
	14	1793.80 ± 60.75 bA	139.63 ± 0.58 aA	798.20 ± 9.17 bA	1743.47 ± 6.15 aAB	355.87 ± 11.98 aA	87.99 ± 1.83 bcB
	15	1791.00 ± 67.26 bA	139.97 ± 1.04 aA	796.53 ± 14.05 bA	1728.93 ± 4.01 abA	359.90 ± 13.60 aA	86.00 ± 2.16 bcB
*p*_Treatment_		<0.001	0.212	<0.001	0.650	<0.001	<0.001
*p*_storage_		0.557	0.436	0.293	<0.001	<0.001	<0.001
*p*_Treatment_*p_storage_		1.000	0.972	0.211	0.344	<0.001	0.002

“Mean ± sd” (*n* = 3) were used to express organic acids concentrations in the Riesling wines. Different lowercase letters represent significant differences in the organic acid concentrations between different groups at the same bottle-storage period. Different capital letters represent significant differences in organic acids concentrations between different bottle-storage durations for the same treatment group (Duncan, *p* < 0.05). *p*_Treatment_, *p*_storage_ and *p*_Treatment_*p_storage_ were the results of two-way ANOVA and showed the differences between different treatment groups and different bottle-storage durations. *p*_Treatment_ was calculated for samples from different cold treatment groups. *p*_storage_ was calculated for samples from different bottle-storage durations. *p*_Treatment_*p_storage_ was calculated from Treatment*bottle storage duration.

## Data Availability

Data are contained within the article or the Supplementary Materials.

## Supplementary Materials

The following supporting information can be downloaded at https://www.mdpi.com/article/10.3390/foods11091179/s1: Table S1: Effects of cold stabilization duration on the concentrations of aroma compounds during wine bottle storage (unit: μg/L); Table S2: The correlation coefficient *r*-values and *p*-values of correlation analyses between the pH value and aroma compounds concentrations, and between different aroma compounds concentrations; Table S3: Effects of cold treatment duration on the odor activity value (OAV) of individual compounds in the Riesling wines during bottle storage [19,22,40,41,42,43,44,45,46,47].
